# Biomarkers of Gut Microbiota in Chronic Spontaneous Urticaria and Symptomatic Dermographism

**DOI:** 10.3389/fcimb.2021.703126

**Published:** 2021-11-09

**Authors:** Runqiu Liu, Cong Peng, Danrong Jing, Yangjian Xiao, Wu Zhu, Shuang Zhao, Jianglin Zhang, Xiang Chen, Jie Li

**Affiliations:** ^1^ Department of Dermatology, Xiangya Hospital, Central South University, Changsha, China; ^2^ Hunan Key Laboratory of Skin Cancer and Psoriasis, Xiangya Hospital, Central South University, Changsha, China; ^3^ Department of Dermatology, The First People’s Hospital of Yancheng, Yancheng, China; ^4^ Department of Dermatology, The Fourth Affiliated Hospital of Nantong University, Nantong, China; ^5^ Department of Dermatology, Hunan Engineering Research Center of Skin Health and Disease, Xiangya Hospital, Changsha, China; ^6^ National Clinical Research Center for Geriatric Disorders, Xiangya Hospital, Changsha, China; ^7^ Xiangya Clinical Research Center for Cancer Immunotherapy, Central South University, Changsha, China

**Keywords:** chronic spontaneous urticaria, symptomatic dermographism, gut microbiota, *Subdoligranulum*, *Ruminococcus bromii*

## Abstract

**Background:**

Chronic urticaria (CU) is a chronic inflammatory skin disease associated with Th2 immune response. The two most common subtypes of CU, i.e., chronic spontaneous urticaria and symptomatic dermographism (CSD), often coexist. However, the pathogenesis of CSD is still unclear. Gut microbiota plays an important role in immune-related inflammatory diseases. The purpose of this study was to explore the correlation between gut microbiota and CSD.

**Methods:**

A case-control study was conducted on CSD patients as well as gender- and age-matched normal controls (NCs). The 16S ribosomal DNA sequencing of fecal samples was used to detect the gut microbiota of all subjects. QPCR was used to further verify the species with differences between the two groups.

**Results:**

The alpha diversity of gut microbiota decreased in CSD patients, accompanied by significant changes of the structure of gut microbiota. *Subdoligranulum* and *Ruminococcus bromii* decreased significantly in CSD patients and had a potential diagnostic value for CSD according to receiver operating characteristic curve (ROC) analysis. *Enterobacteriaceae* and *Klebsiella* were found to be positively correlated with the duration of CSD, while *Clostridium disporicum* was positively correlated with the dermatology life quality index (DLQI).

**Conclusions:**

The gut microbiota of CSD patients is imbalanced. *Subdoligranulum* and *Ruminococcus bromii* are the gut microbiota biomarkers in CSD.

## Introduction

Chronic urticaria (CU), a common recurrent inflammatory skin disease with an incidence rate of 0.1% to 1.4% (Fricke et al., 2020), not only has a serious impact on the work, study, quality of life and mental health of patients, but also imposes a huge economic and social burden to the family and society (Itakura et al., 2018; Zuberbier et al., 2018; Goncalo et al., 2020). However, the pathogenesis of CU is still unknown. According to the 2018 international guidelines for the diagnosis and treatment of urticaria, the diagnosis of CU is mainly based on clinical manifestations and provocation test, and there is still a lack of recognized laboratory diagnostic methods (Zuberbier et al., 2018). The current treatment of CU is largely depending on symptomatic treatment of antihistamines (Zuberbier et al., 2018), but 40%-55% of the patients are ineffective to conventional dose of antihistamines (Guillén-Aguinaga et al., 2016; Joshi and Khan, 2017). Second-line therapy and third-line therapy are immunosuppressants and biological agents respectively, which limit the wide clinical application due to possible serious side effects or expensive price (Zuberbier et al., 2018). Thus, there is an urgent need to find new diagnosis and treatment methods (Amin et al., 2015; Zuberbier et al., 2018).

Previous literature reported that CU was associated with the Th2 cells immune response (Caproni et al., 2004; Kay et al., 2015). Gut microbiota refers to the microorganisms inhabiting the human intestinal tract, which play an important role in regulating the metabolism and immune system of the host (Kundu et al., 2017; Schluter et al., 2020). Up to date, many studies have revealed the potential relationship between gut microbiota and allergic diseases (Wesemann and Nagler, 2016; Hong S et al., 2019; Zimmermann et al., 2019; Galazzo et al., 2020) such as asthma (Fujimura et al., 2016; Barcik et al., 2020; Patrick et al., 2020; Depner et al., 2020), food allergy (Abdel-Gadir et al., 2019; Iweala and Nagler, 2019; Vuillermin et al., 2020), and atopic dermatitis (Avershina et al., 2017; Lee et al., 2018; Mahdavinia et al., 2019). In addition, experimental studies have found that gut microbiota can prevent or treat food allergy (Abdel-Gadir et al., 2019; Feehley et al., 2019) and atopic dermatitis (Avershina et al., 2017).

Recently, a small number of studies have reported an association between gut microbiota and chronic spontaneous urticaria (CSU) or CU (Nabizadeh et al., 2017; Rezazadeh et al., 2018; Lu et al., 2019; Wang et al., 2020). Specifically, CU consists of many subtypes, among which CSU and symptomatic dermographism (SD) are the most common subtypes (Zuberbier et al., 2018). Many subtypes of CU may coexist at the same time; in particular, CSU and SD (CSD) coexist frequently (Zuberbier et al., 2018). However, the relationship between CSD and gut microbiota has not been reported. In this study, we enrolled 25 CSD patients and 25 gender-and age-matched normal controls (NCs). Through the 16S ribosomal gene sequencing of fecal samples, we found that the diversity of gut microbiota in CSD patients decreased, accompanied by significant changes of the structure of gut microbiota. Furthermore, we also found some species with a potential diagnostic value and verified them further by quantitative polymerase chain reaction (qPCR).

## Methods

### Inclusion and Exclusion of Participants

The outpatients and NCs were recruited from the Dermatology Department and the Health Examination Center of Xiangya Hospital, respectively. The diagnosis of CSD was based on the international guidelines for urticaria (Zuberbier et al., 2018).

The inclusion criteria for CSD patients were as follows: 1) meeting the diagnostic criteria of CSU and SD; 2) aged 18-60 years; 3) no autoimmune diseases, gastrointestinal diseases, allergies and other known diseases; 4) no administration of antibiotics, probiotics or prebiotics within 3 months before sample collection; 5) no intake of cheese, yogurt or pickles within 3 days before sample collection; 6) living in Changsha in the past year before sample collection.

The exclusion criteria for CSD patients were as follows: 1) accompanied with other subtypes of urticaria, such as acute urticaria and heat urticaria; 2) unavailability to collect samples as required; 3) pregnancy or lactation.

The inclusion criteria for NCs were as follows: 1) aged 18-60 years; 2) no gastrointestinal diseases, allergic diseases, autoimmune diseases, metabolic diseases or other known diseases (such as hypertension and coronary heart disease); 3) no administration of antibiotics, probiotics or prebiotics within 3 months before sample collection; 4) no intake of cheese, yogurt or pickles within 3 days before sample collection; 5) living in Changsha in the past year before sample collection.

The exclusion criteria for NCs were as follows: 1) unavailability to collect samples as required; 2) pregnancy or lactation.

### Processing and Preservation of Fecal Samples

Fecal samples were processed and preserved according to the following requirements: 1) use a sterile cotton swab to scratch the surface of the stool within 3 minutes after defecation, and then use another sterile cotton swab to extend into the interior of the stool and rotate for 3 circles until there is a piece of feces about the size of soybean on the surface of the cotton swab; 2) put the cotton swab with feces into the collection tube containing DNA preservation solution, and shake the cotton swab gently to make the feces sample evenly dispersed in the preservation solution; 3) put the sample collection tube into the refrigerator at - 80°C for storage.

### DNA Extraction and PCR Amplification

Fecal genomic DNA was extracted by the CTAB/SDS method and the purity of DNA was monitored on 2% agarose gel. DNA was then diluted to 1 ng/μl with sterile water. The v3-v4 region of 16S rRNA gene was amplified by using the following primers combined with barcode: 341F(CCTAYGGGRBGCASCAG), 806R(GGACTACNNGGGTATCTAAT). The PCR reaction was performed with Phusion^®^ High-Fidelity PCR Master Mix (New England Biolabs).

### Library Generation and Sequencing

The sequencing library was generated and the index code was added by using the TruSeq^®^ DNA PCR-Free Sample Preparation Kit (Illumina, USA). The quality of the library was evaluated on the Agilent Bioanalyzer 2100 system and Qubit@ 2.0 Fluorometer (Thermo Scientific). Finally, 250 bp paired-end reads were generated by sequencing on the Illumina NovaSeq6000 platform (Liu et al., 2021).

### Data Analysis

According to the unique barcode of the sample, the paired-end reads were assigned to the sample and combined by FLASH (Magoč and Salzberg, 2011). Following the QIIME (Caporaso et al., 2010) quality control procedure, high quality clean tags were obtained by filtering the raw tags. By comparing the tags and Silva database with the UCHIME (Edgar et al., 2011) algorithm, the chimeric sequences were removed and the effective tags were obtained. Sequences with similarity ≥97% were assigned to the same OTU by Uparse (Edgar, 2013). Based on the Mothur algorithm, the representative sequence of each OTU was annotated with the taxonomic information in the Silva database (Quast et al., 2013). The OTUs abundance normalization was consistent with the sample of the least number of sequences.

Four alpha diversity indices, including Chao1, Observed species, Simpson and Shannon, were used to analyze the alpha diversity. The weighted and unweighted unifrac algorithm was used to analyze the difference of beta diversity among species composition, which was shown in the PCoA diagram. The Wilcoxon rank sum test was used for both groups for numerical variables not conforming to the normal distribution, and the t-test was used for both groups for numerical variables conforming to the normal distribution. The linear discriminant analysis (LDA) effect size (LEfSe) was used to identify the biomarkers with statistical differences between the two groups. The receiver operating characteristic curve (ROC) was used to analyze the potential diagnostic value of specific species. QIIME (Caporaso et al., 2010) software was used to perform core microbiota analysis. Spearman was used to assess the association of gut microbiota with clinical characteristics of the disease, including duration of the disease and dermatology life quality index (DLQI). P < 0.05 indicates a statistically significant difference, unless otherwise specified.

### 16S Ribosomal RNA Gene qPCR

The relative abundance of various species obtained by the 16S ribosomal RNA gene sequencing was further verified by qPCR. The primers for the total bacteria (Maeda et al., 2003; Jian et al., 2020) and Ruminococcus bromii (Mondot et al., 2011) were from different literature (**Table s1**). The specific primers for Subdoligranulum were designed based on the variable region of the 16S ribosomal RNA gene using the Primer-BLAST program (https://www.ncbi.nlm.nih.gov/tools/primer-blast/), and the specificity and coverage were evaluated by the TestPrime 1.0 program (https://www.arb-silva.de, **Table s1**). The annealing temperature and specificity of the primers were verified by gradient PCR.

The qPCR reaction mixture (10ul) was composed of UltraSYBR Mixture (5ul, low Rox), forward and reverse primers (0.2ul) and genomic DNA (5ng). The thermal cycle procedure was as follows: perform initial denaturation at 95°C for 10 min, followed by 40 cycles of the following steps, i.e., denaturation at 95°C for 15 s, annealing at 56°C for 20 s, and extension at 72°C for 1min; then, extend at 72°C for 5 min to end the thermal cycle. After PCR, the melting curve was plotted for the range of 60-95°C. The relative abundance of specific strain (i) was calculated by referencing to the literature (Jian et al., 2020):

Relative abundance (i)=2**^- ΔCT^** =2**^-(CTi-CTt)^**, where CTi and CTt represent the cycling threshold of the strain i primer and the total bacterial primer respectively.

## Results

### Characteristics of Participants

This study included 25 CSD patients and 25 NCs. There were no significant differences in gender, age, waist circumference and body mass index between the two groups (**Table s2**), suggesting that the existing confounding factors did not contribute to the differences between the two groups.

### Analysis of the Diversity of Gut Microbiota

**Figure 1:**
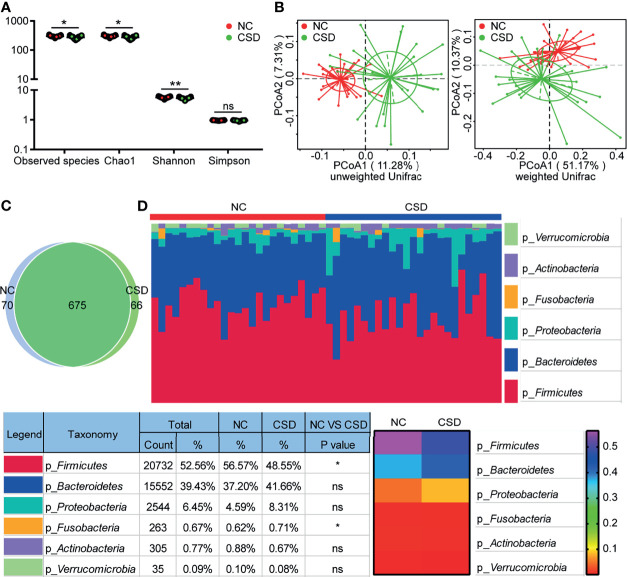
Comparison of alpha diversity, beta diversity, OTU and phylum levels between the CSD group and NC group. **(A)** Three alpha diversity indices (Observed species, Chao1 and Shannon) were lower in the CSD group than in the NC group. **(B)** Based on unweighted unifrac distance and weighted unifrac distance, the CSD group and NC group clustered significantly. **(C)** Common OTUs and unique OTUs in the CSD group and NC group respectively. **(D)** The difference between the CSD group and NC group at the phylum level. NC, normal control; CSD, chronic spontaneous urticaria and symptomatic dermographism. Each point refers to one sample. The sample size of the NC group and CSD group was both 25. The numbers in the bar on the right of the heat map represent the relative abundance of phylum. *p < 0.05, **p < 0.01.

We obtained an average of 69141 raw tags and 414nt base length per sample (**Table s3**) from 16S rRNA gene sequencing. A total of 811 OTUs were annotated in each sample for further analysis. The stable trend of the sparse curves showed that the amount of sequencing of the two groups was enough to cover all the taxa (**Figure s1A**). The stable trend of the species accumulation curves indicated that the applied sample size of the two groups could appropriately reflect the biodiversity (**Figure s1B**). Significant differences were detected in alpha diversity between the two groups for Observed species, Chao1, and Shannon (**Figure 1A**). The difference in beta diversity between the two groups was significant in PCoA plots with both unweighted unifrac distance (Adonis: R^2^ = 0.07, p=0.001) and weighted unifrac distance (Adonis: R^2 ^= 0.07, p=0.016; **Figure 1B**).

### Changes of Gut Microbiota in CSD Patients

Compared with the NC group, the microbial community structure of patients with CSD changed significantly. The CSD group and NC group shared a total of 675 OTUs, while there were 66 and 70 unique OTUs in the CSD group and NC group respectively, according to the Venn diagram (**Figure 1C**). At the phylum level, the proportion of Firmicutes decreased and that of Fusobacteria increased in CSD patients (**Figure 1D**). At the class level, the abundance of Clostridia, Alphaproteobacterial and Deltaproteobacteria decreased, while that of *Gammaproteobacteria* and *Fusobacteria* increased in CSD patients (**Figure s2A**). At the order level, the proportion of *Clostridiales, Rhodospirillales, Caulobacterales* and *Desulfovibrionales* decreased, while that of Enterobacteria and Fusobacteria increased in CSD patients (**Figure s2B**). At the family level, the relative abundance of *Ruminococceae, Rikenellaceae, Muribaculaceae, Christensenellaceae* and *Caulobacteraceae* decreased, while that of *Enterobacteriaceae, Fusobacteraceae, Peptostreptococcaceae* and *Streptococcaceae* increased in CSD patients (**Figure s3**).

### Alterations of Bacterial Taxa and Candidate Markers

**Figure 2:**
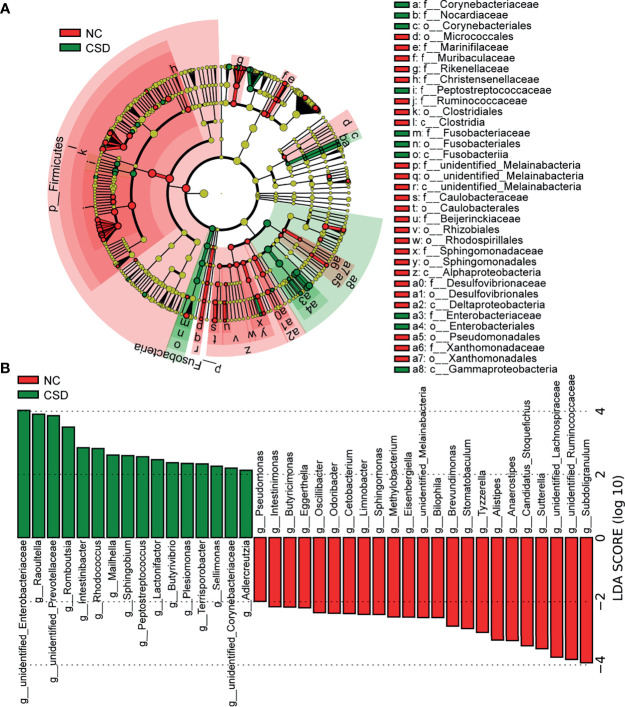
Potential diagnostic markers obtained by linear discriminant analysis (LDA). **(A)** Taxon with a difference between the CSD group and NC group from the class to the genus level. Each small circle represents a different taxonomic level, and the diameter of the small circle is proportional to the relative abundance. Coloring principle: the species with no significant difference are colored yellow uniformly, and those with a significant difference are colored with the group. The red node indicates the microbial groups that play an important role in the NC group, and the green node indicates the microbial groups that play an important role in the CSD group. **(B)** Genus with a significant difference between the CSD group and NC group using the histogram of LDA distribution (p<0.05, LDA scores(log10)>2). NC, normal control; CSD, chronic spontaneous urticaria and symptomatic dermographism.

Candidate biomarkers for patients with CSD were identified by LEfSe (P <0.05, LDA scores (log10)>2). As shown in the LDA *cladogram, Firmicutes, Fusobacteria, Alphaproteobacteria* and *Gammaproteobacteria*, as well as their subordinate taxa, are the main differential taxa between CSD patients and NCs (**Figure 2A**). At the genus level, the relative abundance of 23 genera decreased and that of 16 genera increased in CSD patients (**Figure 2B**). At the species level, the relative abundance of 31 species decreased and that of 15 species increased in CSD patients (**Figure s4**, **Table s4**).

**Figure 3:**
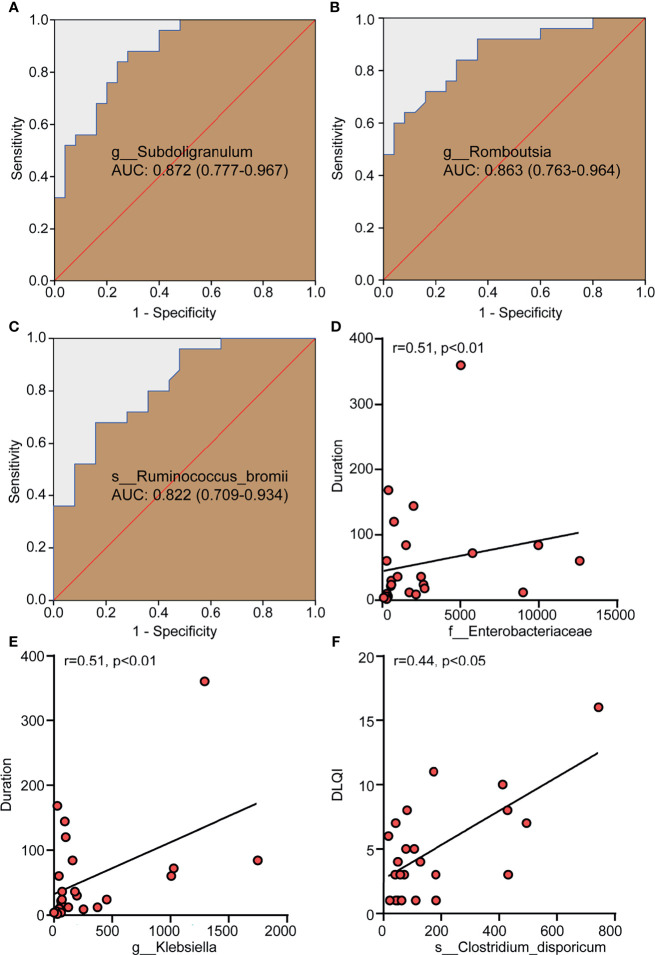
Differential bacteria with a diagnostic value for CSD and gut microbiota taxa with a correlation with clinical parameters. **(A–C)** The potential value of the receiver operating characteristic curve (ROC) analysis based on the bacterial OTU number for correct diagnosis of CSD. **(D, E)** The positive correlation between Enterobacteriaceae and Klebsiella and the duration of CSD. **(F)** The positive correlation between *Clostridium dispericum* and DLQI. r, related coefficient;. NC, normal control; CSD, chronic spontaneous urticaria and symptomatic dermographism; AUC, area under the curve, expressed as AUC value + 95% confidence interval; DLQI, dermatology life quality index.

The abundance of different genera or species was used to evaluate their value in correctly distinguishing CSD patients and HCs through ROC analysis. The areas under the curves (AUC) of Subdoligranulum (**Figure 3A**), *Romboutsia* (**Figure 3B**) and *Ruminococcus bromii* (**Figure 3C**) were 0.872, 0.863 and 0.822 respectively, suggesting that these bacteria have potential diagnostic value in CSD.

Spearman analysis was used to determine the correlation between the relative abundance of Enterobacteriaceae and Klebsiella and the duration of disease (month) and DLQI in patients with CSD. The results showed that the relative abundance of Enterobacteriaceae and Klebsiella were positively correlated with the duration of CSD (**Figure 3D, E**), suggesting that the higher the relative abundance of Enterobacteriaceae and Klebsiella in gut microbiota, it means that the longer the duration of CSD patients. CU, including CSU and SD, had a significant impact on the quality of life of patients. To evaluate the quality of life of the patients with CSD, DLQI was adopted as a widely-used indicator and a high DLQI score corresponded to a poor quality of life (Jáuregui et al., 2011). The results showed that the relative abundance of *Clostridium dispericum* was positively correlated with DLQI (**Figure 3F**), suggesting that the higher the relative abundance of *Clostridium disporicum*, it means that patients with CSD have a poor quality of life.

### Analysis of Functional Core Microbiota

**Figure 4:**
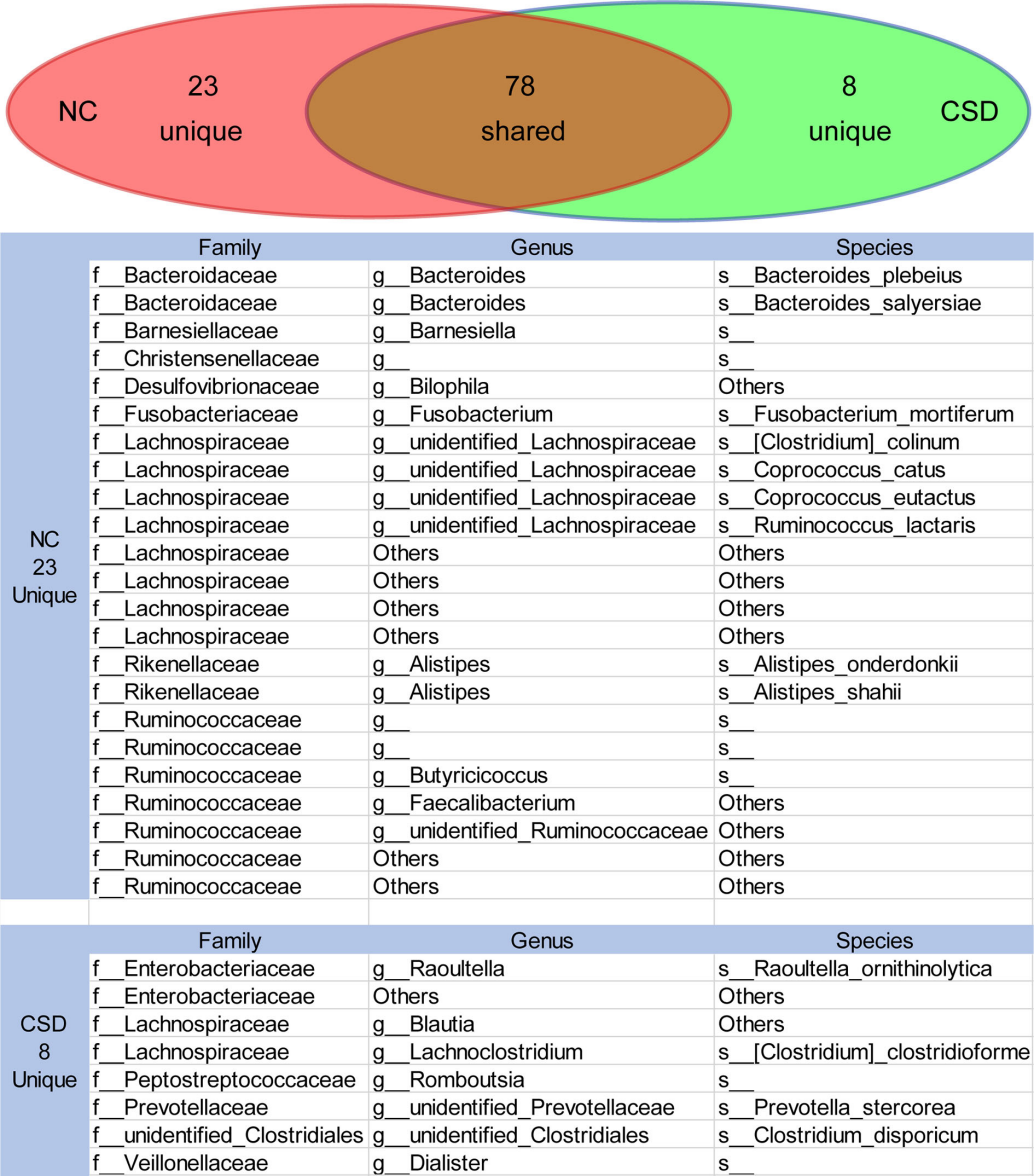
The shared or unique OTUs between the NC group and CSD group were in the core microbiota analysis. Core microbiota is defined as OTUs that exist in more than 90% of individuals with an average relative abundance greater than 0.1%. CSD, chronic spontaneous urticaria and symptomatic dermographism; NC, normal control.

Core microbiome refers to the relatively high abundance of microorganisms shared by the microbial populations with similar habitats (Zhang et al., 2015; Falony et al., 2016). Gut microbiota exists in the form of interacting groups and may function as core groups (Zhang et al., 2015; Kokou et al., 2019). Therefore, the core microbiota would be screened if the relative abundance was greater than 0.1% and shared by more than 90% of the subjects. The results showed that 78 OTUs were shared by the two groups, while there were 8 and 23 unique OTUs in the CSD group and NC group respectively (**Figure 4**). The species or genera of *Bacteroidaceae, Rikenellaceae* and *Ruminocaceae* were relatively scarce in the CSD group, while the species or genera of *Enterobacteriaceae* were relatively abundant in the CSD group (**Figure 4**).

### Changes of Short Chain Fatty Acid Producing Bacteria in Core Microbiota

**Figure 5:**
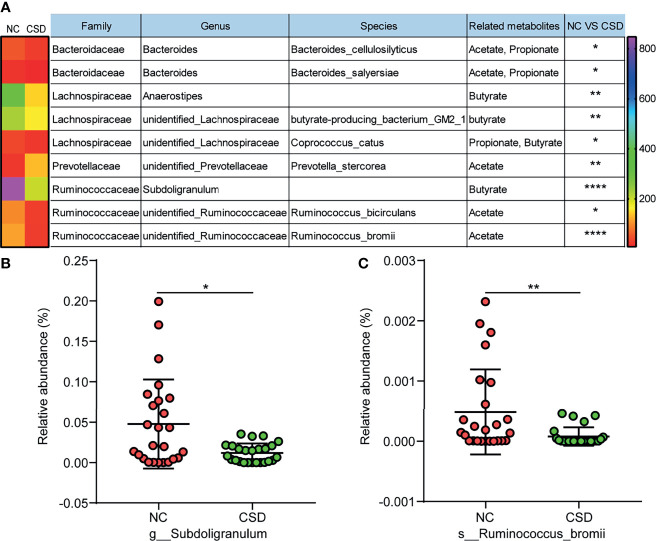
Comparison of short chain fatty acid producing bacteria between the CSD group and NC group. **(A)** Differential short chain fatty acid producing bacteria in core microbiota between the CSD group and NC group. **(B, C)** Differential short chain fatty acid producing bacteria that are further confirmed by qPCR. NC, normal control; CSD, chronic spontaneous urticaria and symptomatic dermographism. The numbers in the bar on the right of the heat map represent the number of OTUs. *p < 0.05, **p < 0.01, ****p < 0.0001.

Metabolites are the key substances in the interaction between gut microbiota and the host, and short chain fatty acids are important metabolites of beneficial bacteria (Koh et al., 2016). Therefore, the short chain fatty acid producing bacteria were analyzed in this study. Among all the short chain fatty acid producing bacteria in the core microbiota, most of them had a decreased relative abundance in CSD patients (**Figure 5A**). Furthermore, some of the short-chain fatty acid producing bacteria with high relative abundance were verified by qPCR, and the results showed that *Subdoligranulum* and *Ruminococcus bromii* decreased in CSD patients (**Figure 5B, C**).

### Hypothetical Model of Dysbacteriosis Involved in the Pathogenesis of CSD

**Figure 6:**
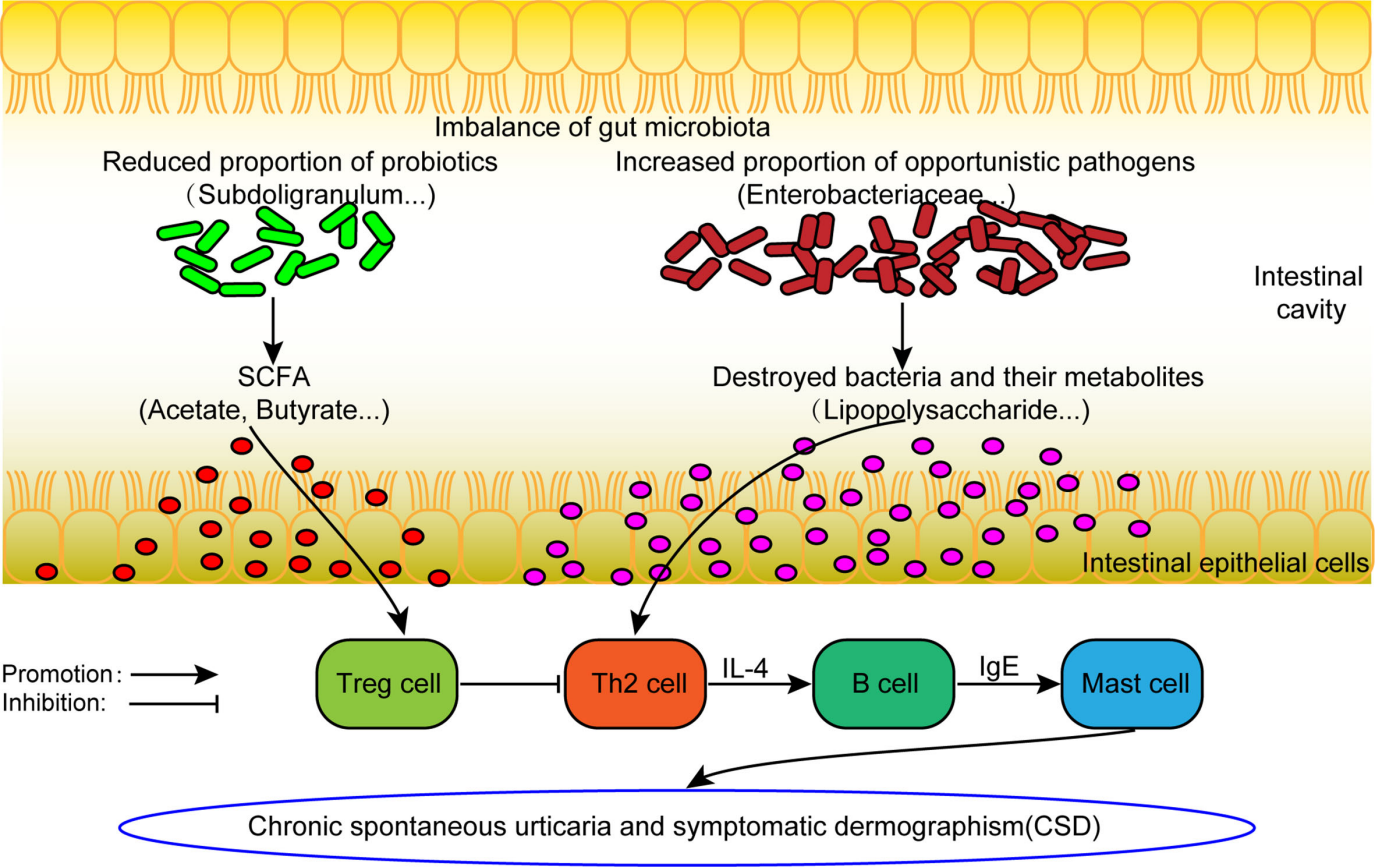
The hypothesis model of dysfunctional gut microbiota involved in the pathogenesis of CSD. SCFA: short chain fatty acid. Treg cell, Regulatory T cell.

From the comprehensive analysis, it was found that beneficial bacteria (mainly short chain fatty acid producing bacteria) decreased in CSD patients, while conditional pathogenic bacteria increased. The results above provide support to the following assumptions (**Figure 6**). On the one hand, the abundance of beneficial bacteria (mainly short-chain fatty acid-producing bacteria) decreases, resulting in a reduction in short-chain fatty acid production, which can in turn decrease the number and weaken the function of regulatory T (Treg) cells (Furusawa et al., 2013). Consequently, the differentiation of initial T cells into Th2 cells can no longer be inhibited. Th2 cells can promote the production of IgE and ultimately activate mast cells to participate in the pathogenesis of CSD. On the other hand, conditional pathogens (such as Enterobacteriaceae), which can produce harmful metabolites such as lipopolysaccharide, are increased. Lipopolysaccharide can promote the differentiation of Th2 cells (Iwasaki et al., 2017), which can further promote the production of IgE by B cells, leading to the activation of mast cells to participate in the pathogenesis of CSD ultimately.

## Discussion

In this study, we found that the alpha diversity of gut microbiota was significantly decreased in CSD patients compared with NCs, resulting in alteration of the community composition. Lu et al. and Wang et al. reported that the alpha diversity of gut microbiota decreased and the composition of gut microbiota changed in patients with CU (no specific subtype was identified) (Lu et al., 2019) and CSU (Wang et al., 2020). The results suggested that the gut microbiota community composition had similar alteration in different types of CU, including CSU and CSD.

From the phylum to the family level, Firmicutes (phylum), Fusobacteria (phylum), Gamma Proteobacteria (class) and their subordinate taxa are the main sources of differences between CSD patients and NCs. Our results are similar to those of Wang et al., who found that the abundance of Firmicutes was significantly reduced in patients with CSU (Wang et al., 2020). In contrast, Lu et al. found that Bacteroidetes was significantly decreased and Actinobacteria and Proteobacteria were increased in patients with CU (Lu et al., 2019). The inconsistency in the results of different studies may be attributed to different subtypes of CU. Firmicutes and Bacteroidetes are the dominant beneficial bacteria in the human intestine (Ley et al., 2006). Their members account for more than 90% of all phylogenetic types in both human and mice (Turnbaugh et al., 2006). The members of Firmicutes in the human gut can degrade insoluble dietary fibers to promote the release of nutrients to other community members and cultivate other species (Berry, 2016). Bacteroides regulate the immune system and maintain a diverse intestinal community by interacting with other microorganisms (Russell et al., 2014). Thus, we speculate that the decrease of Firmicutes promotes the pathogenesis of CSD and CSU, while the decrease of Bacteroides promotes the pathogenesis of CU.

Furthermore, we analyzed the genera and species of the following four families, i.e., *Ruminococcaceae, Christensenellaceae, Fusobacteriaceae* and *Enterobacteria*, which were the main sources of differences between the CSD group and NC group by LEFse analysis. The results showed that *Subdoligranulum, Romboutsia* and *Ruminococcus bromii* were significantly different between the two groups and showed a moderate diagnostic value for CSD. However, the qPCR detection and analysis only confirmed that the relative abundance of *Subdoligranulum* and *Ruminococcus bromii* was significantly reduced in CSD patients. It is noteworthy that the bacteria that we detected with a significant difference between the two groups were inconsistent with those reported in previous literature. Wang et al. found that the abundance of Bacteroides, Faecalibacterium and Bifidobacterium decreased while that of unidentified Enterobacteriaceae increased in patients with CSU (Wang et al., 2020). Nabizadeh et al. reported that the relative abundance or detection frequency of *Akkermansia muciniphila, Clostridium leptum*, and *Faecalibacterium prausnitzii* were significantly decreased in patients with CU (Nabizadeh et al., 2017). Rezazadeh et al. found that the relative abundance of Lactobacillus and Bifidobacterium decreased in patients with CU (Rezazadeh et al., 2018). Lu et al. reported that the abundance of *Faecalibacterium prausnitzii, Prevotella copri*, *and Bacteroides* sp. decreased while that of *Escherichia coli* increased in patients with CU (Lu et al., 2019). The reason for this inconsistency may be that the subtypes of CU we studied are different from those reported in the previous literature, although all of them belong to CU (Nabizadeh et al., 2017; Rezazadeh et al., 2018; Lu et al., 2019; Wang et al., 2020). Secondly, it may also be due to the different research methods used. We used 16S rDNA sequencing to detect the different species and genera between NCs and CSD patients, while some previous literatures only used qPCR to detect the differences of specific species between the two groups (Nabizadeh et al., 2017; Rezazadeh et al., 2018). In general, the relative abundance of beneficial bacteria decreased in patients with CU, while that of opportunistic bacteria increased. Through core microbiota analysis, we found that the relative abundance of the subclasses of *Ruminococcus* (family), including but not limited to *Butyricoccus* and *Faecalibacterium*, decreased, while that of the subclasses of Enterobacteriaceae (family) increased in patients with CSD. Thirdly, we analyzed the short chain fatty acid producing bacteria in the core microbiota and found that most of such bacteria were significantly reduced in CSD patients except for unidentified *Prevotellaceae. Subdoligranulum* is an obligate anaerobe which can produce butyrate. The Subdoligranulum genus contains only one species, i.e., the *Subdoligranulum* variable (Holmstrøm et al., 2004). A variety of diseases, including food allergy (Abdel-Gadir et al., 2019), Behçet syndrome (Consolandi et al., 2015) and preeclampsia (Chang et al., 2020), are associated with the absence or low abundance of Subdoligranulum. The Subdoligranulum variable alone can induce RORγt+ Treg cells and reduce the Th2 immune response of food allergy in mice (Abdel-Gadir et al., 2019). Therefore, we speculate that the decreased abundance of Subdoligranulum promotes Th2 response and CSD through decreased Treg cells. *Ruminococcus bromii* is considered the key cornerstone species, which can initiate the degradation of resistant starch and produce the energy and substances used by other bacterial communities (Ze et al., 2012; Venkataraman et al., 2016). Recent studies have found that *Ruminococcus bromii* has a protective effect on food allergy (Bao et al., 2021). Meanwhile, *Ruminococcus bromii* can also produce acetate, which is beneficial to the production of butyrate downstream. Previous studies have shown that butyrate inhibits inflammation and allergic reactions by inducing the differentiation of Treg cells *in vivo* and *in vitro (* Furusawa et al., 2013). In view of the facts above, both *Subdoligranulum* and *Ruminococcus bromii* can promote the differentiation of Treg cells, although through different ways. Therefore, we speculate that the decrease of the abundance of Subdoligranulum and Ruminococcus bromii leads to the decrease of Treg cells, which is involved in the pathogenesis of CSD.

In addition, we also found that conditional pathogens including Enterobacteriaceae (family) and Klebsiella (genus) were positively correlated with the duration of CSD, and *Clostridium dispericum* was positively correlated with DLQI. The role of *Clostridium dispericum* in gut microbiota remains unclear (O’Connor et al., 2018). Both *Enterobacteriaceae* and *Klebsiella* are Gram-negative bacteria, which can produce lipopolysaccharide. Existing studies have shown that lipopolysaccharide can induce nasal hypersensitivity in mice by activating Th2 cells (Iwasaki et al., 2017). These results imply that opportunistic pathogens may also play a role in the pathogenesis of CSD.

Due to the limitation of the number of samples included in this study, we will continue to expand the sample size and conduct further research *in vivo* through fecal microbial transplantation.

In conclusion, our findings suggest that the gut microbiota of CSD patients is dysfunctional. The relative abundance of short chain fatty acid producing bacteria decreased and that of opportunistic pathogens increased in CSD patients. *Subdoligranulum* and *Ruminococcus bromii* may act as potential markers for the diagnosis of CSD. *Enterobacteriaceae* and *Klebsiella* have a potential predictive value for disease duration, and *Clostridium dispericum* has a potential predictive value for DLQI. Our study provides a new perspective for the diagnosis and intervention of CSD in the future.

## Data Availability Statement

The datasets presented in this study can be found in online repositories. The names of the repository/repositories and accession number(s) can be found in the article/**Supplementary Material**.

## Ethics Statement

The studies involving human participants were reviewed and approved by the Ethics Committee of Xiangya Hospital (Approval number 201904112). The patients/participants provided their written informed consent to participate in this study.

## Author Contributions

RL did the experiment and wrote the manuscript. CP supervised the experiment and analyzed the data. DJ and YX collected data and did bioinformatics analysis. WZ, SZ, and JZ recruited participants and provided necessary tools. XC and JL did the research design and provided the necessary experimental equipment. All authors contributed to the article and approved the submitted version.

## Funding

This study was supported by grants from the National Natural Science Foundation of China (81974476, 81830096, 81773341, 82073458 and 82173424). This study also was supported by Leading talents of scientific and technological innovation in Hunan (2021RC4013), the Program of Introducing Talents of Discipline to Universities (111 Project, No. B20017).

## Conflict of Interest

The authors declare that the research was conducted in the absence of any commercial or financial relationships that could be construed as a potential conflict of interest.

## Publisher’s Note

All claims expressed in this article are solely those of the authors and do not necessarily represent those of their affiliated organizations, or those of the publisher, the editors and the reviewers. Any product that may be evaluated in this article, or claim that may be made by its manufacturer, is not guaranteed or endorsed by the publisher.
