# Supplementary material for: Biomarkers of Gut Microbiota in Chronic Spontaneous Urticaria and Symptomatic Dermographism
Source: Front Cell Infect Microbiol. 2021 Nov 9;11:703126. doi: 10.3389/fcimb.2021.703126 (PMC8630658; doi:10.3389/fcimb.2021.703126)
Supplement: Supplementary file 1 [file DataSheet_1.pdf]

Figure s1

A

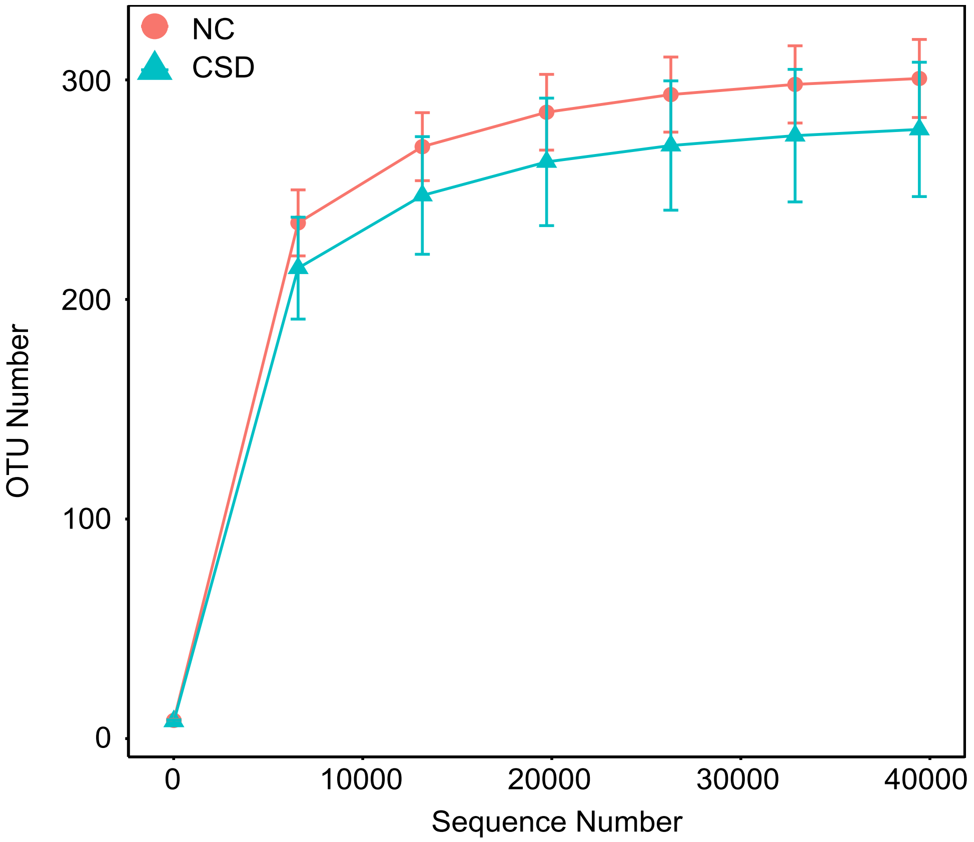

NC
CSD
OTU Number
300
200
100
0
0
10000
20000
30000
40000
Sequence Number

B

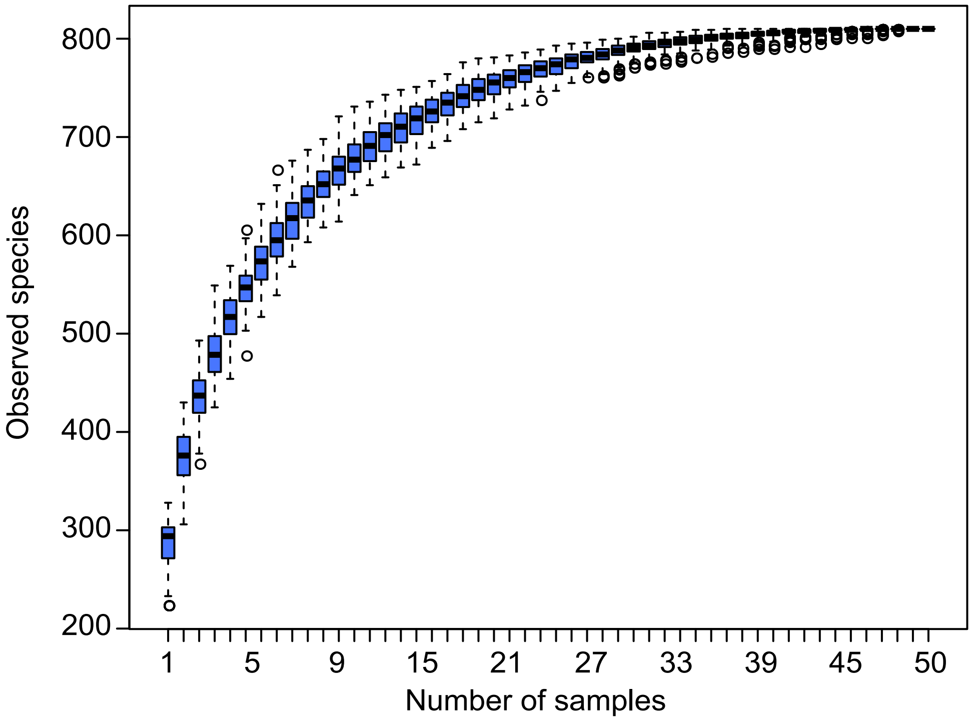

Observed species
800
700
600
500
400
300
200
1
5
9
15
21
27
33
39
45
50
Number of samples

Figure s2

A

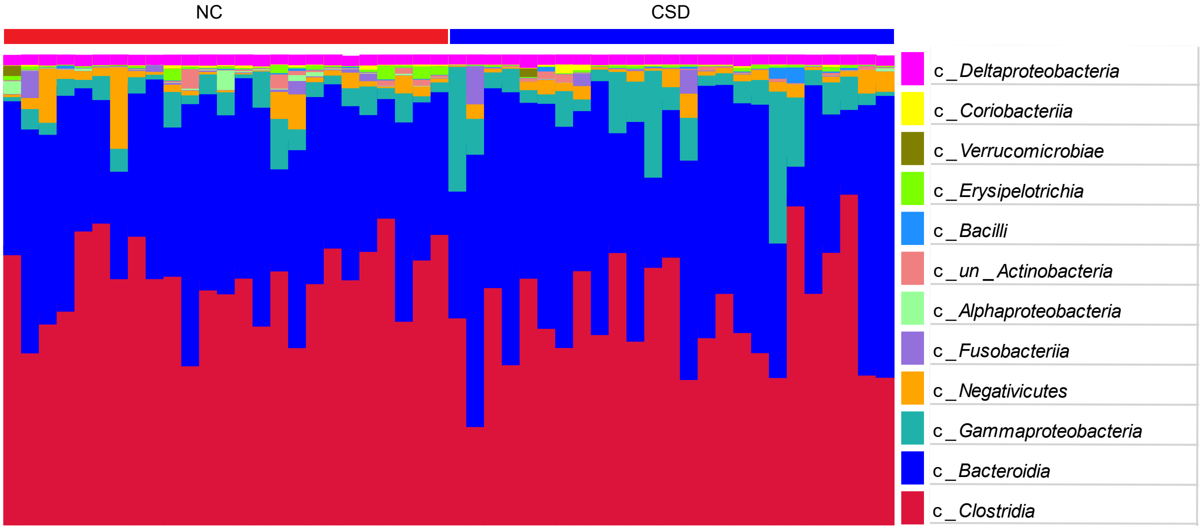

Legend	Taxonomy	Total		NC	CSD	NC VS CSD
		Count	%	%	%	P value
	c_Clostridia	19442	49.29%	52.80%	45.78%	*
	c_Bacteroidia	15552	39.43%	37.20%	41.66%	ns
	c_Gammaproteobacteria	2387	6.05%	3.94%	8.16%	*
	c_Negativicutes	961	2.44%	2.85%	2.02%	ns
	c_Fusobacteriia	263	0.67%	0.62%	0.71%	*
	c_Alphaproteobacteria	105	0.27%	0.49%	0.04%	****
	c_un_Actinobacteria	200	0.51%	0.62%	0.39%	ns
	c_Bacilli	105	0.27%	0.15%	0.38%	ns
	c_Erysipelotrichia	225	0.57%	0.77%	0.37%	ns
	c_Verrucomicrobiae	35	0.09%	0.10%	0.08%	ns
	c_Coriobacteriia	105	0.27%	0.25%	0.28%	ns
	c_Deltaproteobacteria	52	0.13%	0.15%	0.11%	*

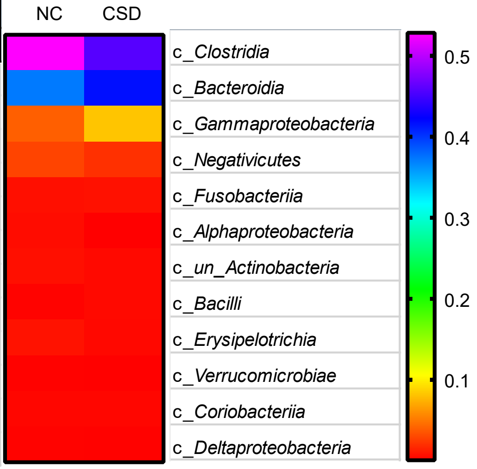

B

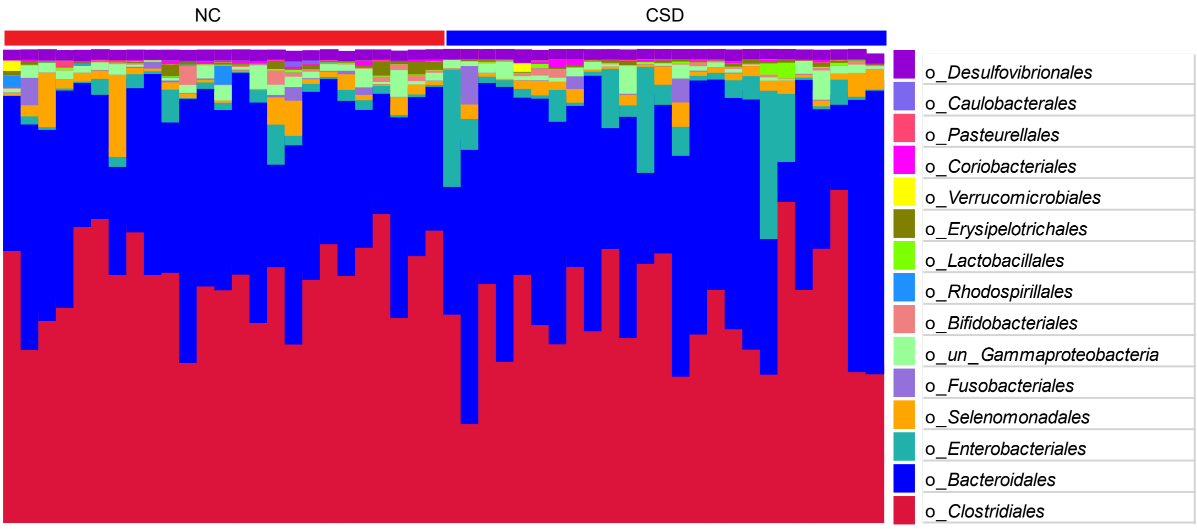

Legend	Taxonomy	Total		NC	CSD	NC VS CSD
		Count	%	%	%	P value
	o_Clostridiales	19442	49.29%	52.80%	45.77%	*
	o_Bacteroidales	15552	39.43%	37.20%	41.66%	ns
	o_Enterobacteriales	1624	4.12%	1.84%	6.40%	*
	o_Selenomonadales	961	2.44%	2.85%	2.02%	ns
	o_Fusobacteriales	263	0.67%	0.62%	0.71%	*
	o_un_Gammaproteobacteria	714	1.81%	1.95%	1.67%	ns
	o_Bifidobacteriales	194	0.49%	0.61%	0.38%	ns
	o_Rhodospirillales	62	0.16%	0.31%	0.00%	*
	o_Lactobacillales	101	0.26%	0.14%	0.38%	ns
	o_Erysipelotrichales	225	0.57%	0.77%	0.37%	ns
	o_Verrucomicrobiales	35	0.09%	0.10%	0.08%	ns
	o_Coriobacteriales	105	0.27%	0.25%	0.28%	ns
	o_Pasteurellales	40	0.10%	0.12%	0.08%	ns
	o_Caulobacterales	24	0.06%	0.12%	0.00%	****
	o_Desulfovibrionales	52	0.13%	0.15%	0.11%	*

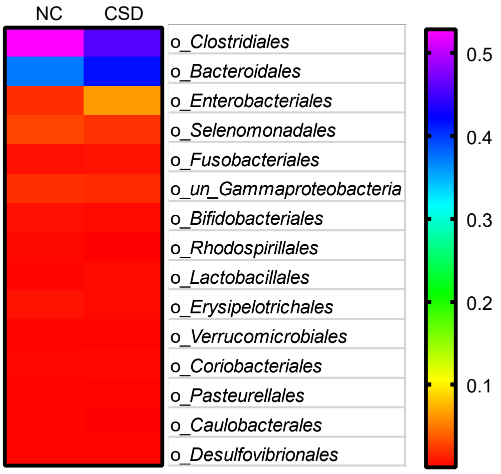

Figure s3

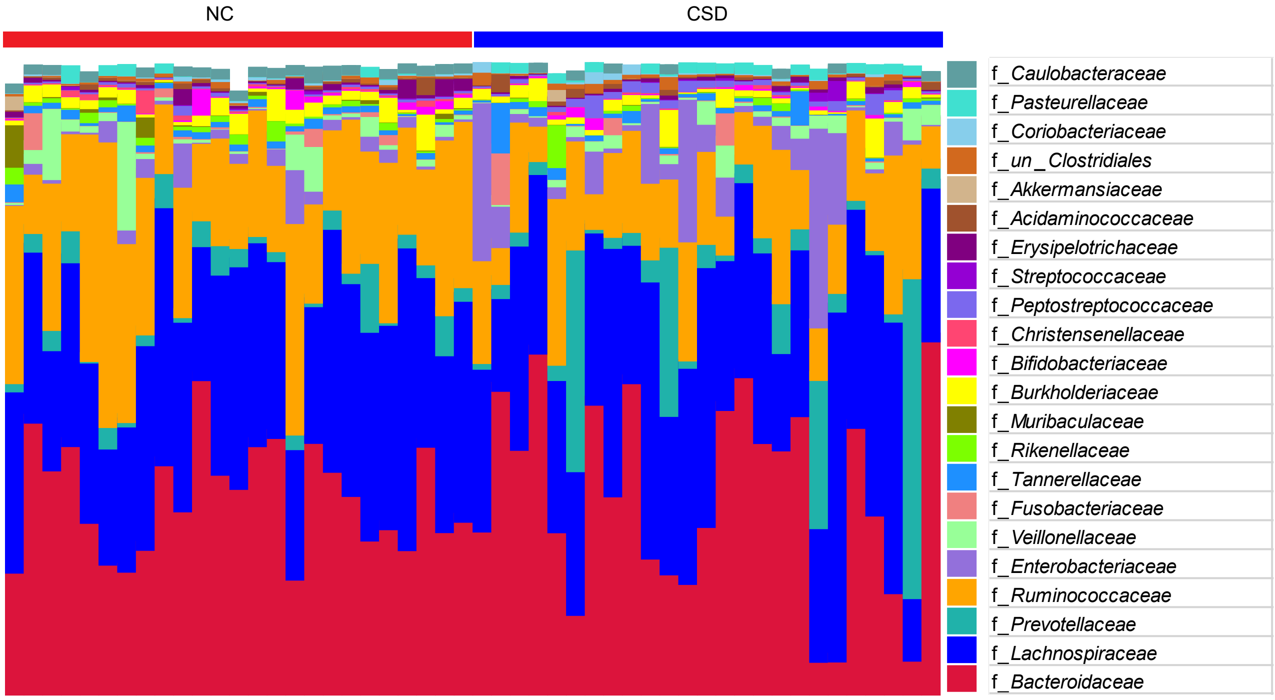

Legend	Taxonomy	Total		NC	CSD	NC VS CSD
		Count	%	%	%	P value
	f_*Bacteroidaceae*	12553	31.82%	31.58%	32.07%	ns
	f_*Lachnospiraceae*	11791	29.89%	29.95%	29.84%	ns
	f_*Prevotellaceae*	2043	5.18%	2.85%	7.51%	ns
	f_*Ruminococcaceae*	7026	17.81%	21.64%	13.99%	***
	f_*Enterobacteriaceae*	1624	4.12%	1.84%	6.40%	*
	f_*Veillonellaceae*	785	1.99%	2.42%	1.56%	ns
	f_*Fusobacteriaceae*	263	0.67%	0.62%	0.71%	*
	f_*Tannerellaceae*	470	1.19%	1.10%	1.29%	ns
	f_*Rikenellaceae*	287	0.73%	0.85%	0.61%	**
	f_*Muribaculaceae*	121	0.31%	0.58%	0.03%	***
	f_*Burkholderiaceae*	711	1.80%	1.94%	1.66%	ns
	f_*Bifidobacteriaceae*	194	0.49%	0.61%	0.38%	ns
	f_*Christensenellaceae*	115	0.29%	0.45%	0.14%	**
	f_*Peptostreptococcaceae*	289	0.73%	0.35%	1.11%	****
	f_*Streptococcaceae*	365	0.93%	0.11%	0.27%	****
	f_*Erysipelotrichaceae*	225	0.57%	0.77%	0.37%	ns
	f_*Acidaminococcaceae*	176	0.45%	0.43%	0.46%	ns
	f_*Akkermansiaceae*	35	0.09%	0.10%	0.08%	ns
	f_*un_Clostridiales*	201	0.51%	0.37%	0.65%	ns
	f_*Coriobacteriaceae*	90	0.23%	0.21%	0.25%	ns
	f_*Pasteurellaceae*	40	0.10%	0.12%	0.08%	ns
	f_*Caulobacteraceae*	22	0.06%	0.11%	0.00%	****

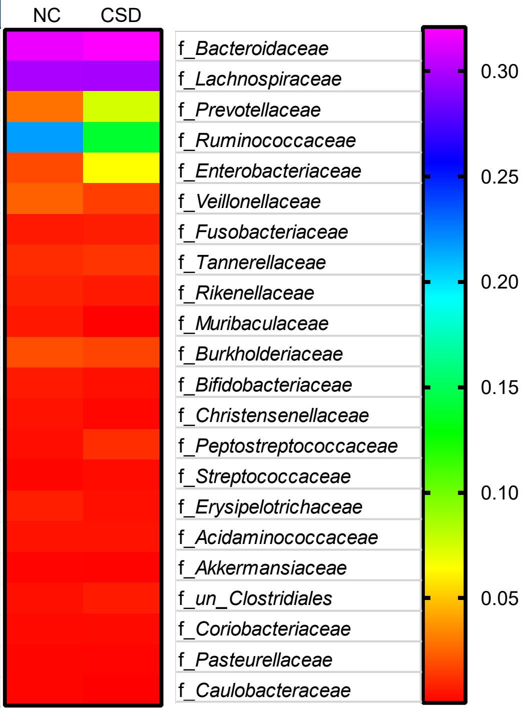

Figure s4

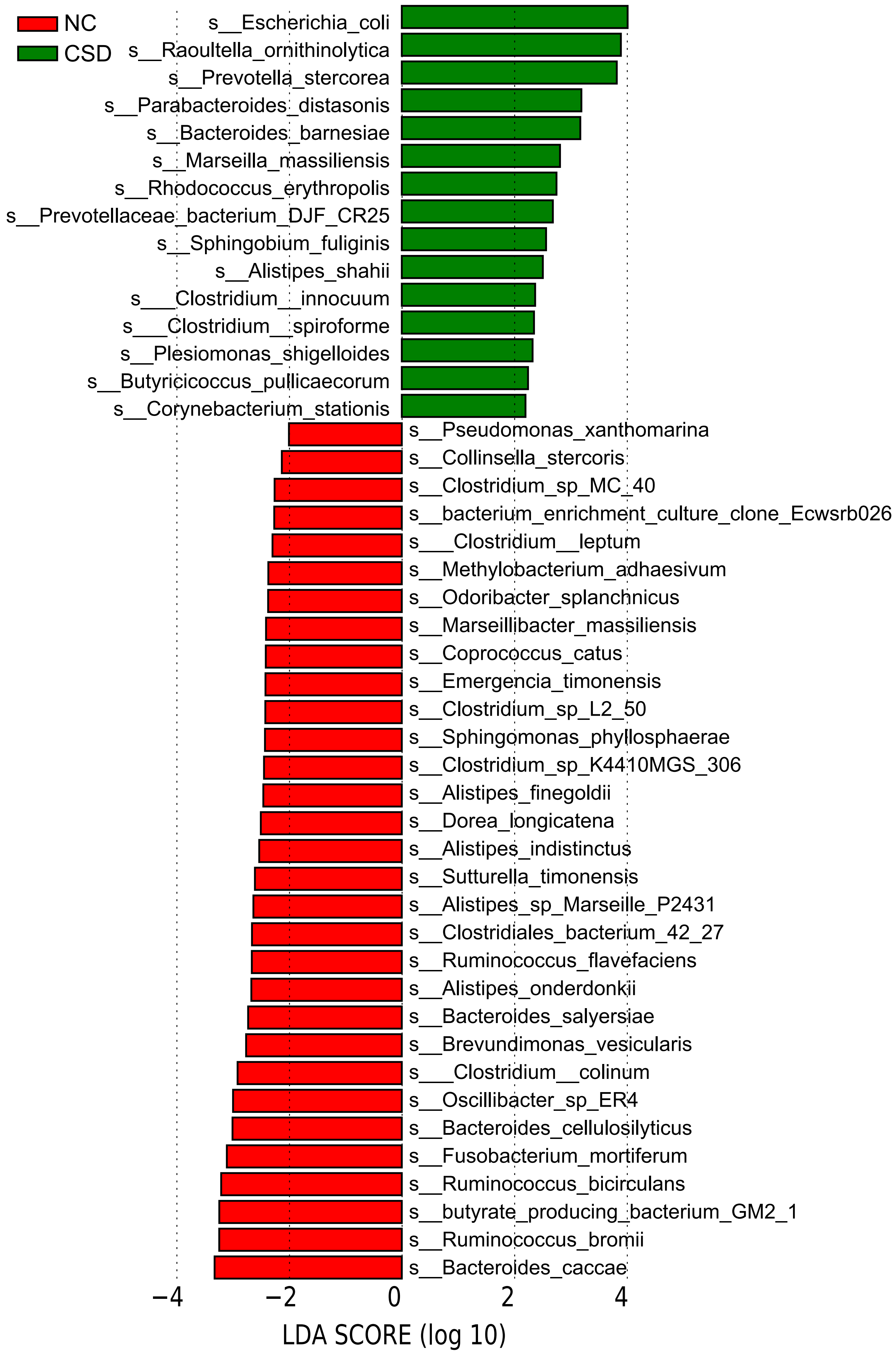
NC
CSD
s__Escherichia_coli
s__Raoultella_ornithinolytica
s__Prevotella_stercorea
s__Parabacteroides_distasonis
s__Bacteroides_barnesiae
s__Marseilla_massiliensis
s__Rhodococcus_erythropolis
s__Prevotellaceae_bacterium_DJF_CR25
s__Sphingobium_fuliginis
s__Alistipes_shahii
s___Clostridium__innocuum
s___Clostridium__spiroforme
s__Plesiomonas_shigelloides
s__Butyricicoccus_pullicaecorum
s__Corynebacterium_stationis
s__Pseudomonas_xanthomarina
s__Collinsella_stercoris
s__Clostridium_sp_MC_40
s__bacterium_enrichment_culture_clone_Ecwsrb026
s___Clostridium__leptum
s__Methylobacterium_adhaesivum
s__Odoribacter_splanchnicus
s__Marseillibacter_massiliensis
s__Coprococcus_catus
s__Emergencia_timonensis
s__Clostridium_sp_L2_50
s__Sphingomonas_phyllosphaerae
s__Clostridium_sp_K4410MGS_306
s__Alistipes_finegoldii
s__Dorea_longicatena
s__Alistipes_indistinctus
s__Sutturella_timonensis
s__Alistipes_sp_Marseille_P2431
s__Clostridiales_bacterium_42_27
s__Ruminococcus_flavefaciens
s__Alistipes_onderdonkii
s__Bacteroides_salyersiae
s__Brevundimonas_vesicularis
s___Clostridium__colinum
s__Oscillibacter_sp_ER4
s__Bacteroides_cellulosilyticus
s__Fusobacterium_mortiferum
s__Ruminococcus_bicirculans
s__butyrate_producing_bacterium_GM2_1
s__Ruminococcus_bromii
s__Bacteroides_caccae
−4
−2
0
2
4
LDA SCORE (log 10)
