# Supplementary material for: Biomarkers of Gut Microbiota in Chronic Spontaneous Urticaria and Symptomatic Dermographism
Source: Front Cell Infect Microbiol. 2021 Nov 9;11:703126. doi: 10.3389/fcimb.2021.703126 (PMC8630658; doi:10.3389/fcimb.2021.703126)
Supplement: Supplementary file 2 [file Table_1.docx]

**Supplementary materials**

| **Table s1 A list of primers used in this study** | | | | |
| --- | --- | --- | --- | --- |
| Taxonomy | Primer | Sequence (5′-3′) | Coverage (%) | Specificity (%) |
| Ruminococcus bromii | Forward primer | GGTCTTGACATCCAACTAACGAAGT | 10 | 100 |
|  | Reverse primer | TTTTGTCAACGGCAGTCCTAT |  |  |
| Subdoligranulum | Forward primer | TGAGTAACCTGCCCTGGAGT | 33.6 | 100 |
|  | Reverse primer | CGAGTCCATCCTGAAGCGAA |  |  |
| Total Bacteria^†^ | Forward primer | GTGSTGCAYYGGYTGTCGTCA | 73.8 | —— |
|  | Reverse primer | ACCGTCRTCCMCACCTTCCTC |  |  |
| †：Total bacterial refers to the primers for amplifying all bacteria. | | | | |

| **Table s2 Characteristics of patients with both chronic spontaneous urticaria and symptomatic dermographism (CSD) and normal controls (NCs)** | | | |
| --- | --- | --- | --- |
| Clinical characteristics | NCs | CSD | NCs VS CSD |
|  | （n=25) | （n=25) | statistical significance |
| Gender (male/female) | 10/15 | 10/15 | ns^§^ |
| Age (years)^†^ | 36.8±9.7 | 36.0±11.8 | ns |
| Waist circumference（cm)^†^ | 77.6±7.6 | 80.4±11.0 | ns |
| Body Mass Index (BMI)^†^ | 23.3±2.6 | 22.6±3.4 | ns |
| Duration of the disease (months)^‡^ | — | 24(9.5-78) | — |
| Dermatology life quality index (DLQI)^‡^ | — | 4(1-7.5) | — |
| †: mean ± standard deviation; ‡: median (25% percentile-75% percentile); ¶: no significance.   \| **Table s3 Average statistics of data output** \| \| \| \| \| \| \| \| \| --- \| --- \| --- \| --- \| --- \| --- \| --- \| --- \| \| Raw PE^†^ \| Raw Tags \| Effective Tags \| Avglen(nt)^‡^ \| Q20 \| Q30 \| GC% \| Effective%^§^ \| \| 71249 \| 69141 \| 63334 \| 414 \| 97.7 \| 93.0 \| 51.2 \| 89.0 \| \| †: the original PE reads off the machine; ‡: the average length of Effective Tags; §: the percentage of Effective Tags in Raw PE. \| \| \| \| \| \| \| \| | | | |

| **Table s4 All species increased or decreased in the CSD group compared with the normal control group.** | |
| --- | --- |
| Increased species | Decreased species |
| Alistipes_shahii Bacteroides_barnesiae Butyricicoccus_pullicaecorum Clostridium__innocuum Clostridium__spiroforme Corynebacterium_stationis Escherichia_coli Marseilla_massiliensis Parabacteroides_distasonis Plesiomonas_shigelloides Prevotella_stercorea Prevotellaceae_bacterium_DJF_CR25 Raoultella_ornithinolytica Rhodococcus_erythropolis Sphingobium_fuliginis | Alistipes_finegoldii Alistipes_indistinctus Alistipes_onderdonkii Alistipes_sp_Marseille_P2431 bacterium_enrichment_culture_clone_Ecwsrb026 Bacteroides_caccae Bacteroides_cellulosilyticus Bacteroides_salyersiae Brevundimonas_vesicularis butyrate_producing_bacterium_GM2_1 Clostridiales_bacterium_42_27 Clostridium__colinum Clostridium__leptum Clostridium_sp_K4410MGS_306 Clostridium_sp_L2_50 Clostridium_sp_MC_40 Collinsella_stercoris Coprococcus_catus Dorea_longicatena Emergencia_timonensis Fusobacterium_mortiferum Marseillibacter_massiliensis Methylobacterium_adhaesivum Odoribacter_splanchnicus Oscillibacter_sp_ER4 Pseudomonas_xanthomarina Ruminococcus_bicirculans Ruminococcus_bromii Ruminococcus_flavefaciens Sphingomonas_phyllosphaerae Sutturella_timonensis |

**Figure Legends**

**Figure s1.** (A) The number of OTUs that can be identified increased with the increase of the depth of sequencing. (B) The species diversity increased accordingly with the increase of the number of samples. CSD: chronic spontaneous urticaria and symptomatic dermographism; NC: normal control.

**Figure s2.** (A) The difference analysis of gut microbiota between the NC group and CSD group at the class level. (B) The difference analysis of gut microbiota between the NC group and CSD group at the order level. CSD: chronic spontaneous urticaria and symptomatic dermographism; NC: normal control. The sample size of the NC group and CSD group was both 25. *p < 0.05, ****p < 0.0001.

**Figure s3.** The difference analysis of gut microbiota between NC group and CSD group at the family level. CSD: chronic spontaneous urticaria and symptomatic dermographism; NC: normal control. The sample size of NC group and CSD group was both 25. *p < 0.05, **p < 0.01, ***p < 0.001, ****p < 0.0001.

**Figure s4.** The taxa with significant differences between the NC group and CSD group were analyzed by the linear discriminant analysis (LDA) effect size at the species level. LDA scores (log10)>2, p<0.05. CSD: chronic spontaneous urticaria and symptomatic dermographism; NC: normal control.
